# The Canines

**Published:** 1867-05

**Authors:** 


### THE CANINES.

Both the little dogs ours and the *Dental Times*—are doing well. Snarler is still able to give a feeble yelp at regular *times;* and Nip is the saucy head of a young family of terriers, which start life with this advantage over Snarler, that their genealogy is not "unknown."

